# Comparison of pressure- and volume-controlled ventilation during laparoscopic colectomy in patients with colorectal cancer

**DOI:** 10.1038/s41598-019-53503-9

**Published:** 2019-11-18

**Authors:** Sangbong Choi, So Young Yang, Geun Joo Choi, Beom Gyu Kim, Hyun Kang

**Affiliations:** 1 Department of Internal Medicine, Division of Respirology, Sanggye Paik Hospital, Inje University College of Medicine, Seoul, Korea; 2 Anesthesiology and Pain Medicine, Seoul, Korea; 3 Department of Surgery, Chung-Ang University College of Medicine, Seoul, Korea

**Keywords:** Predictive markers, Colon cancer, Predictive markers

## Abstract

This study investigated the differences in airway mechanics and postoperative respiratory complications using two mechanical ventilation modalities and the relationship between biomarkers and postoperative respiratory complications in patients with colorectal cancer who underwent laparoscopic colectomy. Forty-six patients with colorectal cancer scheduled for laparoscopic colectomy were randomly allocated to receive mechanical ventilation using either volume-controlled ventilation (VCV) (*n* = 23) or pressure-controlled ventilation (PCV) (*n* = 23). Respiratory parameters were measured and plasma sRAGE and S100A12 were collected 20 minutes after the induction of anesthesia in the supine position without pneumoperitoneum (T1), 40 minutes after 30° Trendelenburg position with pneumoperitoneum (T2), at skin closure in the supine position (T3), and 24 hours after the operation (T4). The peak airway pressure (Ppeak) at T2 was lower in the PCV group than in the VCV group. The plateau airway pressures (Pplat) at T2 and T3 were higher in the VCV group than in the PCV group. Plasma levels of sRAGE at T2 and T3 were 1.6- and 1.4-fold higher in the VCV group than in the PCV group, while plasma S100A12 levels were 2.6- and 2.2-fold higher in the VCV group than in the PCV group, respectively. There were significant correlations between Ppeak and sRAGE, and between Ppeak and S100A12. There were also correlations between Pplat and sRAGE, and between Pplat and S100A12. sRAGE and S100A12 levels at T2 and T3 showed high sensitivity and specificity for postoperative respiratory complications. Postoperative respiratory complications were 3-fold higher in the VCV group than in the PCV group. In conclusion, during laparoscopic colectomy in patients with colorectal cancer, the peak airway pressure, the incidence of postoperative respiratory complications, and plasma sRAGE and S100A12 levels were lower in the PCV group than in the VCV group. Intra- and postoperative plasma sRAGE and S100A12 were useful for predicting the development of postoperative respiratory complications.

## Introduction

Colorectal cancer is the fourth most frequently diagnosed cancer and the second leading cause of cancer death in industrialized countries^1^. In the surgical management of colorectal cancer, laparoscopic colectomy has become a therapeutic option^2^. Several randomized trials and meta-analyses have shown a modest survival advantage, faster recovery, and shorter hospital stays with laparoscopic surgery^3–8^.

However, the insufflation of CO_2_, the most commonly used gas for insufflation, into the peritoneal cavity during laparoscopic surgery can increase the respiratory load due to increased absorption of intraperitoneal CO_2_ and opposition of the diaphragmatic descent due to increased intraperitoneal pressure. This respiratory load is further increased when the patient is in the Trendelenburg position during laparoscopic colectomy^9–11^. While it is not yet clear, such an increase in respiratory load may increase the incidence of postoperative respiratory complications that are significant causes of morbidity and mortality^12,13^. Numerous studies have investigated changes in respiratory parameters according to the mode of mechanical ventilation during laparoscopic surgery. However, there is a lack of studies on the occurrence of postoperative respiratory complications according to the mode of mechanical ventilation applied^14,15^.

Several biomarkers are known to be associated with lung injury. Among them, S100A12 and soluble receptor for advanced glycation end products (sRAGE) are known to increase in the early stage of acute lung injury after surgery^16^. However, the relationship between these biomarkers and postoperative respiratory complications has rarely been studied.

The purpose of the study was to investigate the differences in airway mechanics and postoperative respiratory complications according to the mode of mechanical ventilation during laparoscopic colectomy in patients with colorectal cancer. The secondary objective was to investigate the relationship between biomarkers and postoperative respiratory complications.

## Methods

### Patients

This study was approved by the institutional review board of the Chung-Ang University Hospital (Ref. C2011046[496]) and registered at ClinicalTrials.gov (Ref. NCT02039466) on 17 Jan 2014. This study was performed in accordance with Declaration of Helsinki 2015, and written informed consent was obtained for all participants.

To evaluate differences in respiratory mechanics, plasma biomarkers, and postoperative respiratory complications between pressure-controlled ventilation (PCV) and volume-controlled ventilation (VCV) during laparoscopic colectomy, we investigated consecutive patients with colorectal cancer at a tertiary teaching hospital.

The participants included patients with histologically confirmed colorectal cancers, who were scheduled for laparoscopic colectomy by a single team of surgeons. The patients were 20–65 years old and had an American Society of Anesthesiologists (ASA) physical status score of I to II.

Patients with the following characteristics were excluded: severe cardiovascular, respiratory, renal, or hepatic diseases; psychological disorders; preoperative evidence of infiltration, atelectasis, or pneumonia by chest radiography; history of acute upper or lower respiratory infection requiring medication in the 14 days before the procedure; or patients who refused to be enrolled in the study. Written informed consent was obtained from all participants before being included in the study.

### Randomization and allocation concealment

Enrolled patients were randomly divided into one of two mechanical ventilation modes: the VCV group or the PCV group. The randomization was based on a random table generated using PASS 11 (NCSS, Kaysville, Utah, USA) with Wei’s Urn model. The random sequence was generated by a statistician who was not otherwise involved in the study, and the details of the series were unknown to investigators and patients.

The group assignment was kept in sealed envelopes, and each envelope had only case numbers on the outside. One hour before admitting the patient into the operation room, a blinded investigator (anesthesiologist) opened the assigned envelope to determine whether the patient would be in the VCV group or the PCV group. The preparation of the mechanical ventilator settings was performed by the investigator who read the assignment card. To prevent surgeons and other investigators from knowing the group assigned to the patient, mechanical ventilator settings were covered with labels indicating case numbers.

### Anesthesia and mechanical ventilation

Anesthetic management was performed using a standard protocol. Patients were pre-medicated with intravenous midazolam (1 to 2 mg) in the preoperative holding area. After the patient was transferred to the operation room and standard monitors were positioned, general anesthesia was induced with intravenous propofol, followed by intravenous rocuronium to facilitate endotracheal tube placement. General anesthesia was maintained with sevoflurane (2 to 3 volume%), nitrous oxide (1.8 L/min), and O_2_ (1.2 L/min). Rocuronium was administered, as required, to maintain adequate surgical relaxation. Standard physiological monitoring included electrocardiograph, pulse oximetry, and noninvasive arterial blood pressure measurements. In addition, radial arterial and jugular venous catheters were placed after induction of anesthesia.

The same mechanical ventilator (Aestiva/5, Datex-Ohmeda, GE Healthcare, Chalfont St. Giles, Buckinghamshire, United Kingdom) was used in all subjects. In the VCV group, the ventilation mode was volume-controlled during the entire operation: a tidal volume of 8 mL/kg of ideal body weight (IBW), 0.4 of FiO_2_, respiratory rate of 15 breaths/min, inspiratory–to-expiratory ratio of 1:2, pause time of 0.5 seconds, no positive end-expiratory pressure (PEEP) and 50% of driving pressure. In the PCV group, the initial ventilation mode was volume-controlled (the same setting of volume-controlled ventilation) and after CO_2_ pneumoperitoneum was created, the ventilator mode was changed to pressure-controlled and the inspiratory airway pressure was set and adjusted to deliver a tidal volume of 8 mL/kg of IBW. After decompression of CO_2_ gas, the ventilator mode was changed again to volume-controlled. Ketorolac 30 mg was administered just before the end of the procedure.

### Surgical procedure

The same team of surgeons performed all operations. Standard procedures for laparoscopic colectomy were used. In all patients, access to the peritoneal cavity was established through a 2-cm umbilical incision. CO_2_ pneumoperitoneum was created using an insufflation pressure of 15 mm Hg at a maximum flow rate of 2 L/min, which was restricted electronically during the creation of the pneumoperitoneum and at later stages of the procedure. Laparoscopic colectomy was performed in the lithotomy position at a 20-degree Trendelenburg incline. All patients were managed on a strictly controlled protocol with regard to bowel preparation, antibiotic prophylaxis, analgesic administration, blood transfusion, feeding, and postoperative recovery.

### Measurement of study variables

Preoperatively, ASA physical status, body mass index (BMI), PaO_2_, PaCO_2_, and carcinoembryonic antigen (CEA) were checked. Pulmonary function tests (PFT) were performed during the preoperative evaluation according to the American Thoracic Society guidelines^17^. The duration of anesthesia was defined as the time from injection of propofol to extubation. During the operation, total fluid administered, urine output, expected blood loss, and location (colon/rectum), size, and stage of tumor were recorded.

To monitor respiratory mechanics during the operation, a spirometry module (S/5TM Compact Anesthesia Monitor; Datex-Ohmeda, Tewksbury, MA, USA) was attached to the endotracheal tube. Peak inspiratory airway pressure (Ppeak), plateau airway pressure (Pplat), mean airway pressure (Pmean), dynamic lung compliance (Cdyn), and static lung compliance (Cstat) were recorded by the spirometry module. Pplat in PCV was measured at the point when airflow drops to zero at the end of inspiratory phase before exhalation valve opens. This point could be reached by extending the inspiratory time sufficiently. Measurements of the respiratory mechanics were taken 20 minutes after the induction of anesthesia in the supine position without pneumoperitoneum (T1), 40 minutes after 30° Trendelenburg position with pneumoperitoneum (T2), and at skin closure in the supine position (T3). Each respiratory mechanic parameter was measured 3 times to obtain the mean value, and the value was used as a single measurement. Arterial blood gas analysis was taken at the same time intervals as the respiratory mechanics.

The alveolar-arterial oxygen difference (A-aDO_2_) was calculated and alveolar oxygen tension (P_A_O_2_) was estimated from the following equation:

$${{\rm{P}}}_{{\rm{A}}}{{\rm{O}}}_{2}={{\rm{F}}}_{{\rm{i}}}{{\rm{O}}}_{2}({{\rm{P}}}_{{\rm{B}}}-{{\rm{PH}}}_{2}{\rm{O}})-[{{\rm{PaCO}}}_{2}-(1.25\times {{\rm{PaCO}}}_{2})]$$

where *F*_*i*_*O*_2_ was the fractional concentration of inspired O_2_ (0.21 when breathing room air); *P*_*B*_ was the barometric pressure (760 mm Hg at sea level); and *PH*_2_*O* was the water vapor pressure (47 mm Hg when air is fully saturated at 37 °C).

The alveolar dead space-to-tidal volume ratio (V_D_/V_T_) was estimated using the following equation:

$${{\rm{V}}}_{{\rm{D}}}/{{\rm{V}}}_{{\rm{T}}}=({{\rm{PaCO}}}_{2}-{{\rm{P}}}_{\rm{\ddot{E}}}{{\rm{CO}}}_{2})/{{\rm{PaCO}}}_{2}({{\rm{P}}}_{\rm{\ddot{E}}}{{\rm{CO}}}_{2}\,{\rm{is}}\,{\rm{the}}\,{\rm{mixed}}\,{\rm{expired}}\,{\rm{partial}}\,{\rm{pressure}}\,{\rm{of}}\,{{\rm{CO}}}_{2})$$

Immediately after patients arrived in the recovery room, the anesthesiologist and the attending nurse determined the post-anesthetic recovery score using a modified Aldrete score. The score evaluated 5 signs: activity, respiration, circulation, consciousness, and color. Scores of 0, 1, or 2 were assigned to each sign depending on its absence or presence. At the end of each evaluation, the scores given to each sign were added together. A total score of 10 indicated a patient in the best possible condition^18^. The duration of recovery room stays, diet day, hospital day, intensive care unit (ICU) admission, O_2_ at discharge and respiratory complications were also checked. Diet day was defined as days from the operation to first sip of water.

The clinical definition of postoperative respiratory complications required that a minimum of 2 criteria be documented for 2 or more days (>48 hours) at any time during the first 6 postoperative days: (1) new cough/sputum production, (2) abnormal breath sounds compared to baseline, (3) body temperature >38.0 °C, (4) chest radiograph documentation of atelectasis or new infiltrate, and/or (5) physician documentation of atelectasis or pneumonia^19^. For 6 days after the surgery, 4 trained members of the surgical staff who were not involved in the study and blinded to the group allocation examined each patients assigned to them once daily and determined the postoperative respiratory complications according to the criteria.

### Determination of sRAGE and S100A12 levels in plasma

For each patient, 2 mL of fresh blood was drawn into a vacuum tube containing EDTA at the following time points: 20 minutes after the induction of anesthesia in the supine position without pneumoperitoneum (T1), 40 minutes after the 30° Trendelenburg position with pneumoperitoneum (T2), at skin closure in the supine position (T3), and 24 hours after the operation (T4). After centrifugation at 3000 rpm for 15 minutes at 4 °C, the plasma was divided into aliquots and frozen at −80 °C until assay. sRAGE and S100A12 levels were measured using commercially available ELISA kits (sRAGE: R&D Systems, Minneapolis, MN, USA; S100A12: Cirulex; Cyclex Co. Ltd., Nagano, Japan), according to the manufacturer’s instructions. The total levels of plasma protein were determined with an autobiochemistry analyzer and used for sRAGE and S100A12 normalization (sRAGEN, sRAGE normalized for total protein; S100A12N, S100A12 normalized for total protein). Laboratory staff was blinded to postoperative respiratory complications, and investigators involved in the interpretation of postoperative respiratory complications were blinded to sRAGE and S100A12 levels.

### Statistical analysis

The primary outcome of this study was the Ppeak. To estimate the group size for the study, a pilot study was conducted for Ppeak in 10 patients who received VCV. The averages of Ppeak at T1, T2, and T3 were 16.2, 25.3, and 18.1 cmH_2_O, respectively. The standard deviations of Ppeak ranged from 1.5 to 3.4 cmH_2_O, and an autocorrelation between adjacent measurements on the same individual was 0.7. For our power calculation, we assumed that first-order autocorrelation adequately represented the autocorrelation pattern. The aim was to detect 10% decrease of Ppeak in the PCV group compared with the VCV group. A 10% reduction of Ppeak was chosen as clinically significant reduction with reference to previous studies^20,21^. With an α of 0.05 and a power of 80%, an estimated 21 patients were needed per group. Considering a 10% follow-up loss, we enrolled 46 patients in this study. The PASS 11™ software (NCSS, Kaysville, UT, USA) was used to calculate the sample size.

We used an intention-to-treat strategy; that is, all enrolled patients were included in the analysis irrespective of whether they completed the study. As some patients had missing data relative to the outcome variables, missing data were completed using the last observation carrying forward (LOCF) method. The Shapiro-Wilk test was used to test for the normality of variables. Normally distributed data were analyzed using a parametric test, and abnormally distributed data were analyzed using a non-parametric test. The age, forced vital capacity (FVC), PaO_2_, urine output, recovery room stay time, PaO_2_/FiO_2_, Vd/Vt, Cdyn, Cstat, and sRAGE passed the Shapiro-Wilk test. The BMI, forced expiratory volume in 1 second (FEV_1_), FEV_1_/FVC, PaCO_2_, anesthetic time, total fluid administered, blood loss, tumor size, CEA, postanesthetic recovery score, hospital day, and diet day did not pass the normality test. In addition, serially checked data, A-aDO_2_, Ppeak, Pplat, Pmean, and S100A12 did not pass the Shapiro-Wilk test, thus, we evaluated a q-q plot, which did not show marked deviation from linearity. Therefore, the normal assumptions were applied for the repeated measured ANOVA.

As PaO_2_/FiO_2_ (χ^2^[2] = 2.482, *p* = 0.269, Mauchly’s W = 0.945), A-aDO_2_ (χ^2^[2] = 2.516, *p* = 0.284, Mauchly’s W = 0.944), Vd/Vt (χ^2^[2] = 2.783, *p* = 0.249, Mauchly’s W = 0.939), Ppeak (χ^2^[2] = 3.127, *p* = 0.209, Mauchly’s W = 0.930), and Pplat (χ^2^[2] = 2.598, *p* = 0.273, Mauchly’s W = 0.941) passed Mauchly’s sphericity test, they were analyzed by Repeated Measures of ANOVA (RM-ANOVA) with a Greenhouse-Geisser correction, followed by paired *t*-test followed by Bonferroni correction (α = 0.05/3 = 0.0167). As Mauchly’s sphericity test indicated that the assumption of sphericity had been violated for Cdyn (χ^2^[2] = 13.853, *p* = 0.001, Mauchly’s W = 0.730), Cstat (χ^2^[2] = 14.121, *p* < 0.001, Mauchly’s W = 0.712), Pmean (χ^2^[2] = 33.299, *p* < 0.001, Mauchly’s W = 0.461), sRAGE (χ^2^[5] = 11.116, *p* = 0.049, Mauchly’s W = 0.771), and S100A12 (χ^2^[5] = 40.992, *p* < 0.001, Mauchly’s W = 0.383), Wilk’s Lambda’s multivariate analysis of variance (MANOVA) followed by paired *t*-test with Bonferroni correction (α = 0.05/3 = 0.0167 or α = 0.05/4 = 0.0125) was used.

The Pearson’s correlation coefficient was used to measure the correlation between Ppeak and sRAGE or S100A12 levels, as well as between Pplat and sRAGE or S100A12. Receiver operating characteristic (ROC) curve analysis was used to determine the cut-off level of sRAGE and S100A12 for predicting postoperative respiratory complications. Descriptive data were analyzed using the chi-square test or the Fisher’s exact test, as appropriate. Normally distributed data were presented as mean ± standard deviation (SD), and abnormally distributed data were presented as median (Q_1_–Q_3_). Data in figures were presented with mean and standard error (SE).

## Results

### Patient characteristics

**Figure 1:**
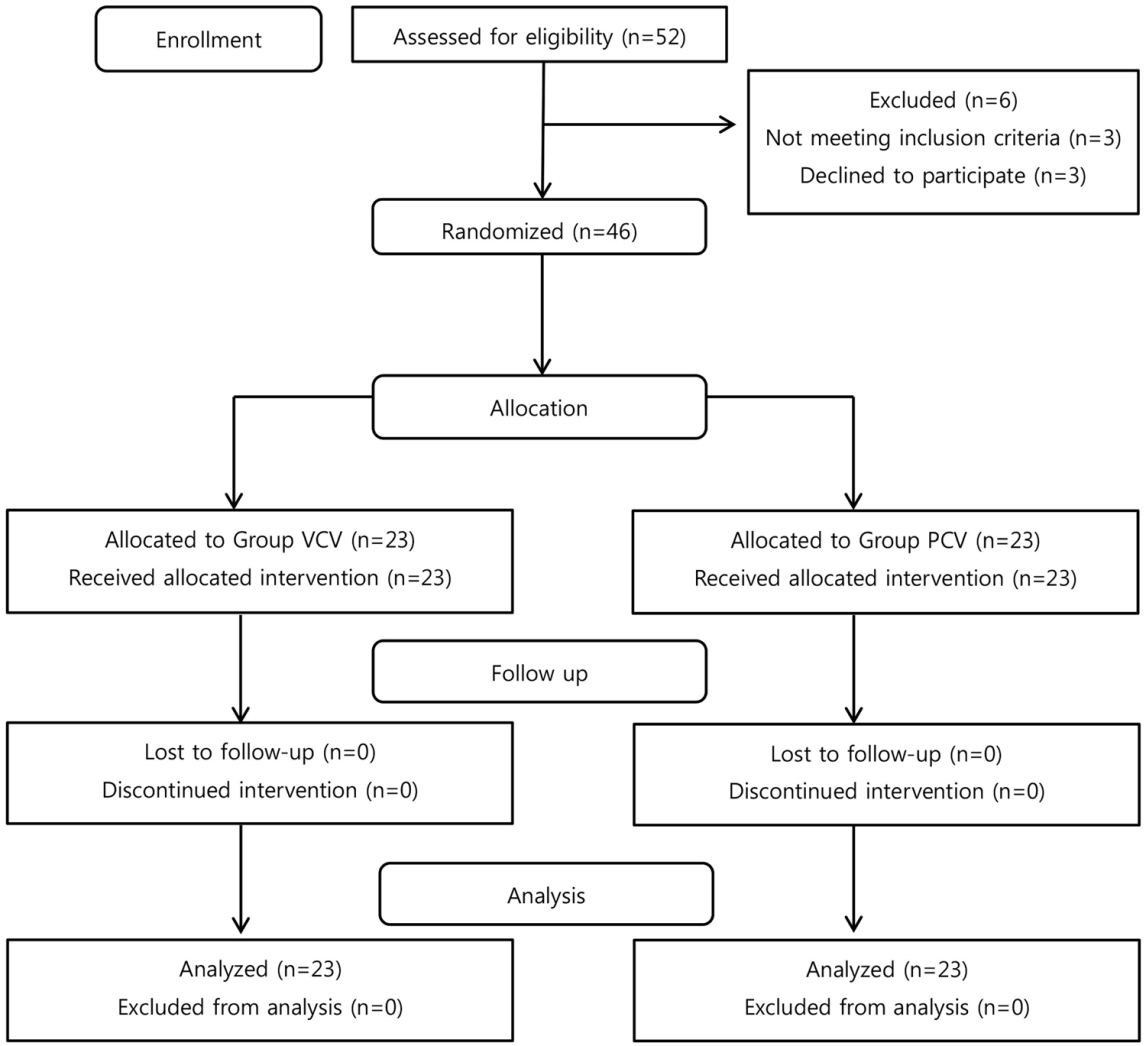
Enrollment and randomization of the study participants.

From January 2014 to December 2015, a total of 52 patients with colorectal cancer were screened. Of these, 46 patients met the inclusion criteria and were included in the study. Of the 46 patients, 23 were assigned to the VCV group and 23 to the PCV group; none of the assigned patients were excluded from the study (Fig. 1). There were no significant differences between the two groups with respect to age, sex, BMI, ASA grade, FVC, FEV_1_, FEV_1_/FVC, PaO_2_, PaCO_2_, A-aDO_2_, the anesthesia duration, the amount of fluid administered, urine output, and blood loss, CEA, and location, size, and stage of tumors (Table 1).

**Table 1:** Demographics and perioperative variables of 46 patients with colorectal cancer.

	VCV group (n = 23)	PCV group (n = 23)	*P* value
Gender (M/F)	14/9	15/8	0.750
Age (yr)	61.1 ± 9.4	65.3 ± 8.6	0.123
BMI (kg/m^2^)	23.2 (21.4–26.2)	23.8 (20.7–26.7)	0.684^a^
ASA I/II	11/12	7/16	0.227
FVC (%)	101.3 ± 27.7	104.0 ± 15.4	0.658
FEV_1_ (%)	108.0 (84.0–138.0)	110.0 (96.0–124.0)	0.982^a^
FEV_1_/FVC	77.0 (75.0–80.0)	73.0 (71.0–79.0)	0.071^a^
PaO_2_ (mm Hg)	94.1 ± 19.9	93.2 ± 13.6	0.850
PaCO_2_ (mm Hg)	38.0 (33.0–45.0)	38.0 (34.0–40.0)	0.473^a^
A-aDO_2_	4.1 ± 15.4	9.7 ± 14.7	0.216
Anesthesia time (min)	220.0 (200.0–250.0)	250.0 (215.0–290.0)	0.285^a^
Total Fluid (mL)	2250.0 (1600.0–2850.0)	2300.0 (2000.0–2800.0)	0.362^a^
Urine output (mL)	587.2 ± 396.4	653.9 ± 819.1	0.727
Blood Loss (mL)	200.0 (150.0–300.0)	150.0 (100.0–200.0)	0.303^a^
CEA (ng/mL)	2.2 (1.6–5.1)	3.7 (2.6–6.6)	0.173^a^
Tumor location (colon/rectum)	22/1	23/0	
Tumor size (cm)	3.0 (2.5–4.5)	3.0 (1.7–3.8)	0.377^a^
Stage (I, II, III, IV)	8/9/4/2	10/5/6/2	0.623

VCV: volume-controlled ventilation, PCV: pressure-controlled ventilation, M: male, F: female, BMI: body mass index, ASA: American Society of Anesthesiologists physical status, FVC: forced vital capacity, FEV_1_: Forced expiratory volume at 1 second, A-aDO_2_: Alveolar-arterial O_2_ difference, Ane: Anesthesia, CEA: carcinoembryonic antigen.

Data are presented mean ± standard deviation, median (Q_1_–Q_3_), or absolute number.

^a^Data are presented as median (Q_1_–Q_3_) and analyzed using Mann-Whitney U test because of abnormal distribution.

### Comparison of respiratory parameters

There were no significant differences between the PCV group and the VCV group with respect to PaO_2_/FiO_2_, and A-aDO_2_ (F [1, 99920.503] = 1.304, *p* = 0.260 and F [1, 9627.718] = 1.177, *p* = 0.284, respectively). The overall Vd/Vt was higher in the VCV group than in the PCV group (F [1, 0.037] = 5.016, *p* = 0.030), but there were no differences at each time point. The MANOVA showed statistically significant differences between the two groups with respect to overall Cdyn and Cstat (F [3.0, 42.0] = 454.648, *p* < 0.001: Wilk’s lambda = 0.030, partial eta^2^ = 0.970 and F [3.0, 44.0] = 471.23, *p* < 0.001: Wilk’s lambda = 0.021, partial eta^2^ = 0.981). The Cdyn of the PCV group was higher at T2 and the Cstat of the PCV group was higher at T2 and T3 compared to those of the VCV group (Table 2).

**Table 2:** Changes of respiratory parameters during laparoscopic colectomy.

	T1	T2=	T3	P value
PaO_2_/FiO_2_				
VCV	564.6 ± 142.2	435.6 ± 150.3	471.7 ± 119.7	0.260
PCV	560.1 ± 139.6	518.6 ± 120.0	513.8 ± 131.9	
A-aDO_2_				
VCV	13.5 ± 69.5	49.6 ± 84.7	20.1 ± 49.4	0.284
PCV	13.2 ± 56.4	22.5 ± 50.6	18.8 ± 26.1	
Vd/Vt				
VCV	0.1 ± 0.1	0.2 ± 0.1	0.2 ± 0.1	0.030
PCV	0.1 ± 0.1	0.1 ± 0.1	0.1 ± 0.1	
Cdyn (ml/cmH_2_O)				
VCV	34.8 ± 8.9	20.6 ± 4.1	29.8 ± 5.7	<0.001
PCV	34.7 ± 7.1	26.3 ± 5.7*	31.8 ± 5.8	
Cstat (ml/cmH_2_O)				
VCV	46.3 ± 11.7	27.6 ± 6.9	41.2 ± 12.5	<0.001
PCV	45.9 ± 10.9	45.9 ± 32.2*	46.1 ± 13.1*	

T1: 20 minutes after the induction of anesthesia in the supine position without pneumoperitoneum, T2: 40 minutes after 30° Trendelenburg position with pneumoperitoneum, T3: at skin closure in the supine position, VCV: volume-controlled ventilation, PCV: pressure-controlled ventilation, A-aDO_2_: Alveolar-arterial O_2_ difference, Vd: dead space volume, Vt: tidal volume, Cdyn: dynamic lung compliance, Cstat static lung compliance.

Data are presented mean ± standard deviation. *p < 0.05 compared with VCV.

### Comparison of airway pressures

**Figure 2:**
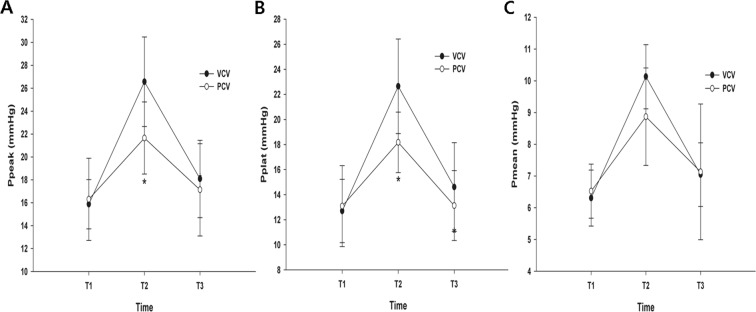
Changes in airway pressure during laparoscopic colectomy. (**A**) Peak airway pressure, (**B**) Plateau airway pressure, (**C**) Mean airway pressure. *p < 0.05 compared to volume-controlled ventilation (VCV).

The Ppeak at T2, the primary end-point, was lower in the PCV group than in the VCV group (26.57 ± 3.67 *vs*. 21.65 ± 2.27 mm Hg) (Fig. 2A). The Pplat at T2 and T3 were higher in the VCV group than in the PCV group (T2: 21.91 ± 4.27 *vs*. 18.17 ± 2.13 mm Hg; T3: 15.17 ± 4.07 *vs*. 13.09 ± 2.78 mm Hg) (Fig. 2B). The overall Ppeak and Pplat were lower in the PCV group than in the VCV group (F [1, 113.225] = 5.710, *p* = 0.021 and F [1, 118.725] = 6.540, *p* = 0.014). However, there were no significant differences between the two groups with respect to Pmean (F [3.0, 42.0] = 1.314, *p* = 0.283: Wilk’s lambda = 0.021, partial eta^2^ = 0.086) (Fig. 2C).

### Comparison of plasma biomarkers

**Figure 3:**
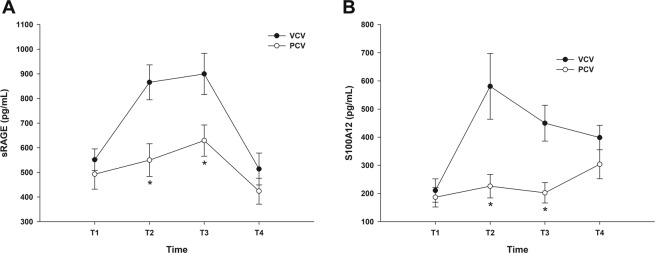
Changes in plasma sRAGE and S100A12 levels during laparoscopic colectomy. (**A**) sRAGE, (**B**) S100A12. *p < 0.05 compared with volume-controlled ventilation (VCV).

The overall plasma levels of sRAGE and S100A12 were higher in the VCV group than in the PCV group (F [4.0, 41.0] = 66.312, *p* < 0.001: Wilk’s lambda = 0.021, partial eta^2^ = 0.866 and F [4.0, 41.0] = 39.869, *p* < 0.001: Wilk’s lambda = 0.205, partial eta^2^ = 0.795). The plasma level of sRAGE at T2 and T3 were 1.6 and 1.4 times higher in the VCV group than in the PCV group (Fig. 3A), while those of plasma S100A12 were 2.6 and 2.2 times higher in the VCV group than in the PCV group, respectively (Fig. 3B).

### Correlation between plasma biomarkers and airway pressures

**Figure 4:**
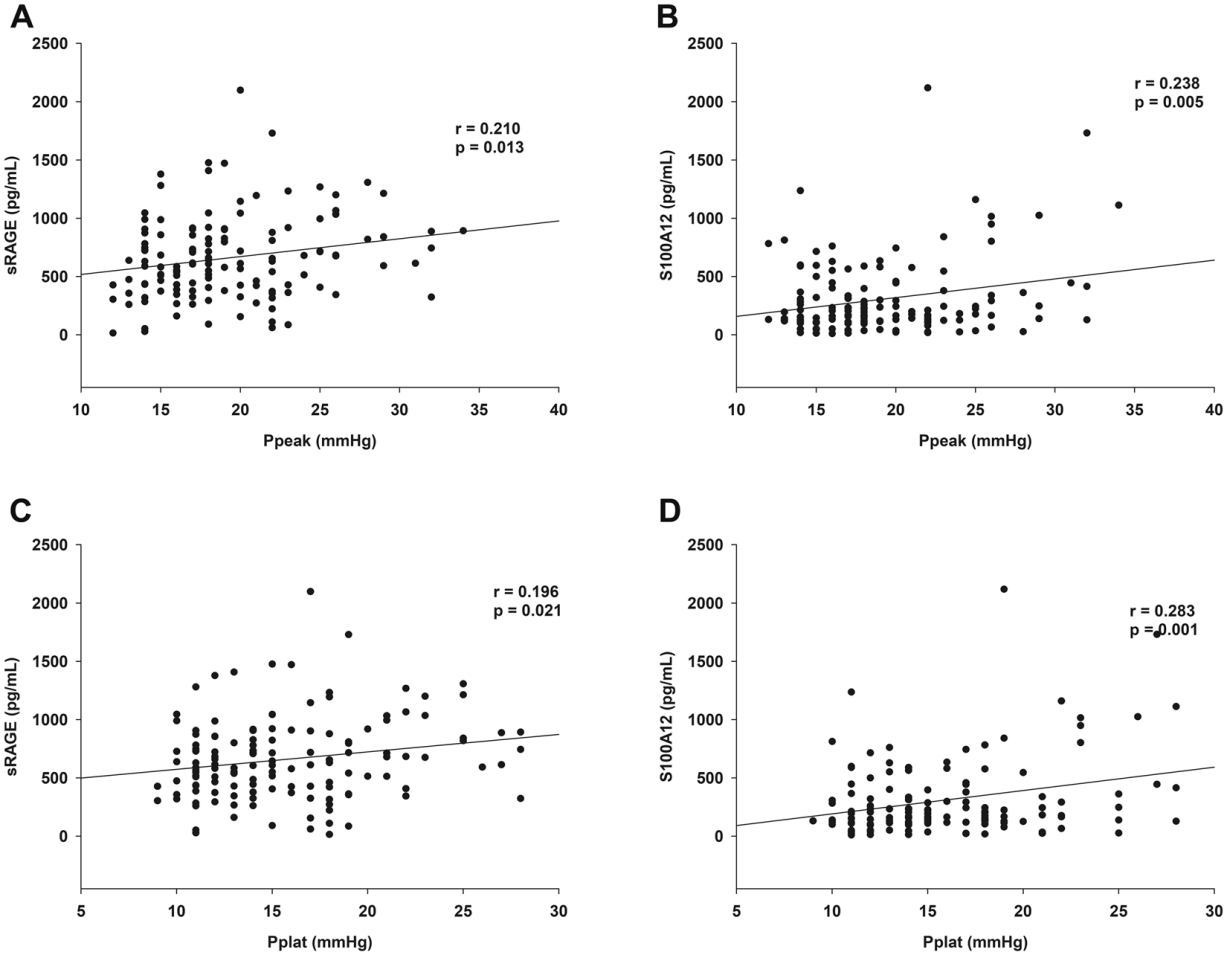
Correlation between biomarkers and airway pressure. (**A**) sRAGE and peak airway pressure, (**B**) S100A12 and peak airway pressure, (**C**) sRAGE and plateau airway pressure, (**D**) S100A12 and plateau airway pressure.

There were significant correlations between Ppeak and sRAGE (r = 0.210, *p* = 0.013), and between Ppeak and S100A12 (*r* = 0.238, *p* = 0.005) (Fig. 4A,B). In addition, there were significant correlations between Pplat and sRAGE (r = 0.196, *p* = 0.021), and between Pplat and S100A12 (*r* = 0.283, *p* = 0.001) (Fig. 4C,D).

### Predictive value of plasma biomarkers for postoperative respiratory complications

**Figure 5:**
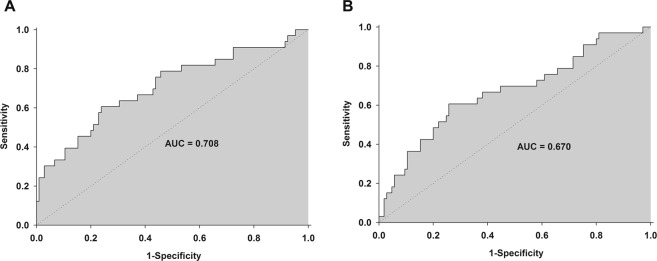
The receiver-operator characteristic analysis. (**A**) sRAGE, (**B**) S100A12.

In the ROC analysis, the AUC of sRAGE and S100A12 at T2 and T3 for postoperative respiratory complications were 0.708 (95% confidence interval [CI] 0.599–0.817) and 0.670 (95% CI 0.559–0.781), respectively (Fig. 5A,B). The optimal cut-off values of sRAGE and S100A12 to predict postoperative respiratory complications were 762.82 mcg/mL (sensitivity 61%, specificity 76%) and 284.25 pg/mL (sensitivity 61%, specificity 64%), respectively.

### Ventilator modes and postoperative respiratory complications

There were no significant differences between the two groups with respect to postoperative characteristics and the incidence of the 5 criteria indicative of postoperative respiratory complications: new cough or sputum, abnormal breath sounds, fever, and atelectasis or infiltrates (as documented by chest radiography or physician). However, postoperative respiratory complications defined as having two or more of the criteria were three times higher in the VCV group than in the PCV group (Table 3).

**Table 3:** Postoperative characteristics and respiratory complications.

	VCV group (n = 23)	PCV group (n = 23)	P value
RR stay time (min)	38.0 ± 17.6	35.9 ± 12.0	0.627
Recovery score	7.0 (6.0–8.0)	7.0 (6.0–8.0)	0.439
Hospital day	10.0 (7.0–13.0)	9.0 (7.0–11.0)	0.293
Diet day	4.0 (3.0–4.0)	4.0 (3.0–4.0)	0.662
ICU admission	1 (4.3)	1 (4.3)	1.000
O_2_ at discharge	9 (39.1)	4 (17.4)	0.102
Chest X-ray atelectasis or infiltrates	9 (39.1)	4 (17.4)	0.102
Physician Atelectasis or pneumonia	5 (21.7)	2 (8.7)	0.218
Fever	4 (17.4)	1 (4.3)	0.155
New cough or sputum	4 (17.4)	3 (13.0)	0.681
Abnormal breath sound	3 (13.0)	1 (4.3)	0.295
≥2 respiratory complications	9 (39.1)	3 (13.0)	0.044*

VCV: volume-controlled ventilation, PCV: pressure-controlled ventilation, RR: recovery room, ICU: intensive care unit.

Data are presented mean ± standard deviation, median (Q_1_–Q_3_), or absolute number (%). * p < 0.05 between groups.

## Discussion

The results from this prospective study showed that among patients with colorectal cancer who had undergone laparoscopic colectomy, the peak airway pressure was lower in the PCV group than in the VCV group. The incidence of postoperative respiratory complications and the plasma levels of sRAGE and A100S12 were lower in the PCV group than in the VCV group. Intra- and postoperative plasma sRAGE and A100S12 were useful for predicting the development of postoperative respiratory complications.

The lower airway pressure observed in PCV than in VCV may be due to differences in inspiratory flow waveforms. The inspiratory flow waveform of PCV exhibits a decelerating pattern, and that of VCV is constant. It is known that decelerating the inspiratory flow waveform may reduce peak airway pressure, respiratory resistance, ratio of dead space to tidal volume (Vd/Vt), and alveolar-arterial gradient for oxygen (A-aDO_2_) and may also increase static and kinetic respiratory compliance compared to the constant inspiratory flow waveform of the VCV^22^. In this study, respiratory compliances were also greater in the PCV group than in the VCV group. In a similar study of patients who had undergone laparoscopic cholecystectomy, the PCV showed in a significant decrease in intraoperative mean peak airway pressure compared to the VCV^14^. However, on the contrary, there are some animal studies showing that high inspiratory flow rate increases ventilator-induced lung injury^23–25^. Because viscoelastic accommodation has insufficient time to dissipate damaging forces when inflation occurs quickly, the high inspiratory flow rate can accentuate damage to the lung parenchyma. But this type of mechanism of injury usually occurs in inhomogenous lungs (e.g., ARDS).

In fact, to determine whether inspiratory flow waveform affected the difference in airway pressure, the airway pressure should have been measured after decelerating waveform was applied to the VCV group, but we did not in this study.

A possible explanation for the one-third lower incidence of postoperative respiratory complications in the PCV group than in the VCV group might be that the pressure-controlled mode could keep the airway pressure lower than the volume-controlled mode, rather than the effect of the ventilation mode itself. It is well known that in patients with acute lung injury and the acute respiratory distress syndrome, mechanical ventilation with a lower tidal volume results in decreased mortality^26^. There is also robust evidence that a lung-protective ventilation strategy with low tidal volumes in patients undergoing major abdominal surgery is protective against postoperative respiratory complications^27^. Accordingly, to reduce the development of postoperative respiratory complications, the setting of the mechanical ventilator should be adjusted to keep the airway pressure low during the surgery. Other previous studies have reported that differences in the incidence of postoperative respiratory complications did not depend on the ventilator modes^28,29^. Recently, a large-scale study showed that postoperative respiratory complications were more common after PCV than VCV. However, the study was conducted retrospectively, and the intraoperative airway pressure could not be maintained at a certain level. PCV delivered more varied and higher driving airway pressures and tidal volume than VCV^30^.

sRAGE and S100A12, biomarkers associated with lung injury, were found to be highly concentrated in pulmonary tissue and bronchoalveolar lavage fluid in acute lung injury and have been known to predict acute lung injury after surgery^16,31,32^. In this study, sRAGE and S100A12 were higher in the VCV group than in the PCV group. It is possible that the volume-controlled mode kept the airway pressure higher than the pressure-controlled mode, and the high airway pressure seemed to increase the biomarkers associated with lung injury.

In this study, sRAGE and S100A12 were associated with respiratory complications and were useful to predict the development of respiratory complications after laparoscopic colectomy. The mechanism for increasing postoperative respiratory complications in patients with increased these biomarkers is not clear. Increased intraoperative airway pressure may cause lung injury, which may lead to inflammatory reactions or respiratory symptoms, leading to postoperative respiratory complications.

Although the study was conducted prospectively in consecutive patients with colorectal cancer in a tertiary teaching hospital, it has several limitations. First, the study was designed to compare the intraoperative airway pressure according to ventilator modes, not to compare postoperative respiratory complications or biomarkers. Although postoperative respiratory complications were lower in the PCV group than in the VCV group, many studies have shown that the mechanical ventilation setting associated with postoperative respiratory complications was the intraoperative airway pressure. Second, sRAGE and S100A12 have been reported to be associated with lung injury rather than with postoperative respiratory complications. Though sRAGE and S100A12 were able to predict postoperative respiratory complications, the mechanism is still unclear. Nevertheless, this study was not designed to identify such mechanisms. Third, biomarkers were measured using plasma samples. It is not known whether the same results would have been obtained had the biomarker levels been measured using lung tissues or bronchoalveolar lavage fluid. Fourth, a European joint taskforce published guidelines for perioperative clinical outcome definition in 2015^33^, however, our study used a relatively simple definition of postoperative respiratory complications^19^. Fifth, the application of PEEP in patients with acute respiratory distress syndrome is clear to reduced lung damage caused by mechanical ventilator, but we did not apply PEEP because this study was conducted with patients undergoing surgery. However, since neither group used PEEP, not using PEEP would not have affected the results of the study.

In patients with colorectal cancer who underwent laparoscopic colectomy, peak airway pressure was lower in the PCV group than in the VCV group. The incidence of postoperative respiratory complications and the plasma level of sRAGE and A100S12 were lower in the PCV group than in the VCV group. Intra- and postoperative plasma sRAGE and A100S12 were useful for predicting the development of postoperative respiratory complications. Further studies should be designed to determine the optimal cut-off value for biomarkers to predict postoperative respiratory complications.
