# Author Correction: Comparison of pressure- and volume-controlled ventilation during laparoscopic colectomy in patients with colorectal cancer

**DOI:** 10.1038/s41598-020-59904-5

**Published:** 2020-03-06

**Authors:** Sangbong Choi, So Young Yang, Geun Joo Choi, Beom Gyu Kim, Hyun Kang

**Affiliations:** 1 Department of Internal Medicine, Division of Respirology, Sanggye Paik Hospital, Inje University College of Medicine, Seoul, Korea; 2 Anesthesiology and Pain Medicine, Chung-Ang University College of Medicine, Seoul, Korea; 3 Department of Surgery, Chung-Ang University College of Medicine, Seoul, Korea

Correction to: *Scientific Reports* 10.1038/s41598-019-53503-9, published online 18 November 2019

The original version of this Article contained an error in Affiliation 2, which was incorrectly given as ‘Anesthesiology and Pain Medicine, Seoul, Korea’. The correct affiliation is listed below:

Anesthesiology and Pain Medicine, Chung-Ang University College of Medicine, Seoul, Korea

This error has now been corrected in the HTML and PDF versions of the Article.

